# Investigating the impact of data heterogeneity on the performance of federated learning algorithm using medical imaging

**DOI:** 10.1371/journal.pone.0302539

**Published:** 2024-05-15

**Authors:** Muhammad Babar, Basit Qureshi, Anis Koubaa

**Affiliations:** 1 Robotics and Internet of Things Lab, Prince Sultan University, Riyadh, Saudi Arabia; 2 College of Computer and Information Sciences, Prince Sultan University, Riyadh, Saudi Arabia; National University of Sciences and Technology NUST, PAKISTAN

## Abstract

In recent years, Federated Learning (FL) has gained traction as a privacy-centric approach in medical imaging. This study explores the challenges posed by data heterogeneity on FL algorithms, using the COVIDx CXR-3 dataset as a case study. We contrast the performance of the Federated Averaging (FedAvg) algorithm on non-identically and independently distributed (non-IID) data against identically and independently distributed (IID) data. Our findings reveal a notable performance decline with increased data heterogeneity, emphasizing the need for innovative strategies to enhance FL in diverse environments. This research contributes to the practical implementation of FL, extending beyond theoretical concepts and addressing the nuances in medical imaging applications. This research uncovers the inherent challenges in FL due to data diversity. It sets the stage for future advancements in FL strategies to effectively manage data heterogeneity, especially in sensitive fields like healthcare.

## 1 Introduction

Federated Learning (FL) is a compelling approach for machine learning in scenarios where data privacy, security, and efficient distributed learning are critical considerations [1, 2]. This concept enables various devices in a distributed environment, each with its local data, to collaboratively train a shared model, generating knowledge of all participating devices, leading to enhanced model performance and generalization. FL allows models to be trained on decentralized data sources, ensuring that sensitive and personal data remains on the user’s device and is not shared centrally. This enhances privacy and data security, making it suitable for applications in healthcare [3–6]. Furthermore, since only model updates are shared rather than raw data, FL significantly reduces the amount of data transmitted over networks, reducing bandwidth requirements.

Although FL shows excellent potential, data heterogeneity issues emerge from the discrepancies in data distributions across various devices in the distributed networks [7, 8]. This variance is quite common in real-world scenarios, often due to the diversity of users’ behaviors or preferences. Consequently, this heterogeneity could highly affect the performance of FL algorithms [9]. The variety in user behaviors can lead to considerable inconsistencies in the local data of different clients, which can precipitate unstable and sluggish model convergence [10] and even lead to below-par or harmful model performance [11]. A vast array of research has been dedicated to uncovering potential solutions to these issues when dealing with non-Independent and Identically Distributed (non-IID) data environment (i.e., heterogeneous) data and unbalanced datasets in FL.

Morafah et.al., [7] report that data heterogeneity in FL impacts the accuracy performance of the participating clients. Li et.al in [8] note that data heterogeneity in FL causes the update direction of some clients to hinder other clients, so the global model makes it difficult to treat each user fairly. They note that the current fair FL methods usually use the variance of model performance to measure fairness, which can not quantify the fairness of the FL process. Researchers in [10] observe that the variety in user behaviors can lead to considerable inconsistencies in the local data of different clients, which can precipitate unstable and sluggish model convergence and even lead to below-par or harmful model performance. Authors in [12] note that data heterogeneity across various clients results in a notable degradation of model performance negatively affecting the accuracy. Yao et.al. [13] coined the term client-model-drift addressing the data heterogeneity. As each client trains the model locally, its local objectives might diverge significantly from the collective goal. As a result, the global model, effectively the mean value of local models, may not align with the global optimum. This consequently culminates in the subpar performance of the global model.

Comprehending the impact of data heterogeneity on convergence holds significant importance. This understanding directly influences the optimization of Federated Learning (FL) algorithms, particularly in practical scenarios where heterogeneity is frequently unavoidable. The detrimental consequences of data heterogeneity present a significant obstacle to the successful deployment of FL in real-world environments [11]. A vast array of research has been dedicated to uncovering potential solutions to these issues when dealing with non-IID data in FL. To this end, recent works by authors in [10] present FedProx, to tackle heterogeneity in federated networks. FedProx is a generalization and re-parametrization of the popular FedAvg algorithm commonly used for federated learning. The authors show that data convergence using the modified FedAvg across a suite of realistic federated datasets improves the test accuracy. Understanding how data heterogeneity impacts convergence is of substantial significance. It has direct implications for optimizing FL algorithms, particularly in real-world settings where heterogeneity is often inevitable. The adverse effect of data heterogeneity poses a considerable challenge for deploying FL in a real environment. While the heterogeneity of data is a crucial aspect of FL, it’s important to note that using pre-built TensorFlow Federated (TFF) datasets like MNIST and CIFAR primarily caters to generic use cases while overlooking the nuances of more specific applications [14]. For instance, we might need to apply more than one dataset when considering a more complex real-world scenario, such as analyzing COVID-19 images from chest X-rays [15–17]. These specific cases often entail more data heterogeneity and complexity, underscoring the importance of designing FL mechanisms to handle such diversity. Table 1 summarizes the introduction section.

**Table 1:** Overview of Federated Learning (FL) and data heterogeneity.

Aspect	Details
Concept	Federated Learning (FL) is a machine learning approach that emphasizes data privacy, security, and efficient distributed learning. It enables devices in a distributed environment to train a shared model collaboratively while keeping the data localized.
Advantages	Enhances model performance and generalization, ensuring data privacy and security. Reduces data transmission over networks, thereby cutting down bandwidth requirements. Particularly beneficial in healthcare contexts.
Challenges	Data heterogeneity, arising from varying data distributions across devices, leads to performance issues in FL algorithms. This includes unstable model convergence and potentially subpar model performance.
Research Focus	Addressing non-Independent and Identically Distributed (non-IID) data environments and unbalanced datasets in FL.
Key Observations	Impact of data heterogeneity on accuracy performance in FL. Data heterogeneity in FL can cause update direction issues, hindering fairness.
FedProx Solution	A generalization of the FedAvg algorithm to tackle data heterogeneity, enhancing data convergence and test accuracy in federated networks.
Research Study	Investigates the impact of data heterogeneity on FL algorithm performance, using COVIDx CXR-3 dataset and other larger datasets to simulate IID and non-IID environments.

This research study investigates the impact of data heterogeneity on the performance of FL algorithms. We developed a methodology that accurately formats data for federated learning, accounting for client counts, communication rounds, and dataset size variations. This method ensures optimal distribution among different clients and establishes a robust foundation for experiments. We explore the impact of data heterogeneity on the performance of FedAvg, a representative FL algorithm, using the COVIDx CXR-3 dataset, partitioned to simulate IID and non-IID environments. Additionally, two other datasets, albeit with larger sizes, are also considered to verify the validity of our approach through extensive simulations. Performance is characterized by the number of communication rounds (epochs), the number of clients, and the choice of the deep learning model, in addition to the final model accuracy parameters, which are the crucial aspects of FL.

Our research provides key insights into the challenges and intricacies of federated learning in the context of data heterogeneity. The contributions of this work include:

- We developed a methodology that accurately formats data for federated learning, accounting for client counts, communication rounds, and dataset size variations. This approach guarantees an efficient allocation among clients and establishes a robust foundation for experiments.
- We conducted an extensive simulation study using COVIDx CXR-3 dataset and similar datasets to study data variability’s impacts on model performance and convergence.
- We provide a comparative analysis of the IID and non-IID environments using the proposed approach. Based on this analysis we provide a clearer perspective on the distinct challenges and outcomes of the proposed approach.

The rest of the paper is organized as follows: Section 2 reviews recent works in FL related to healthcare and data heterogeneity in FL. Section 3 presents the hypothesis and limitations of the proposed work. Section 4 presents the proposed methodology detailing the various aspects of the proposed model. Section 5 presents the results of the experimental study and provides a detailed analysis of the results. Section 6 discusses the potential solutions to mitigate data heterogeneity, followed by conclusions in Section 7.

## 2 Background

**Fig 1:**
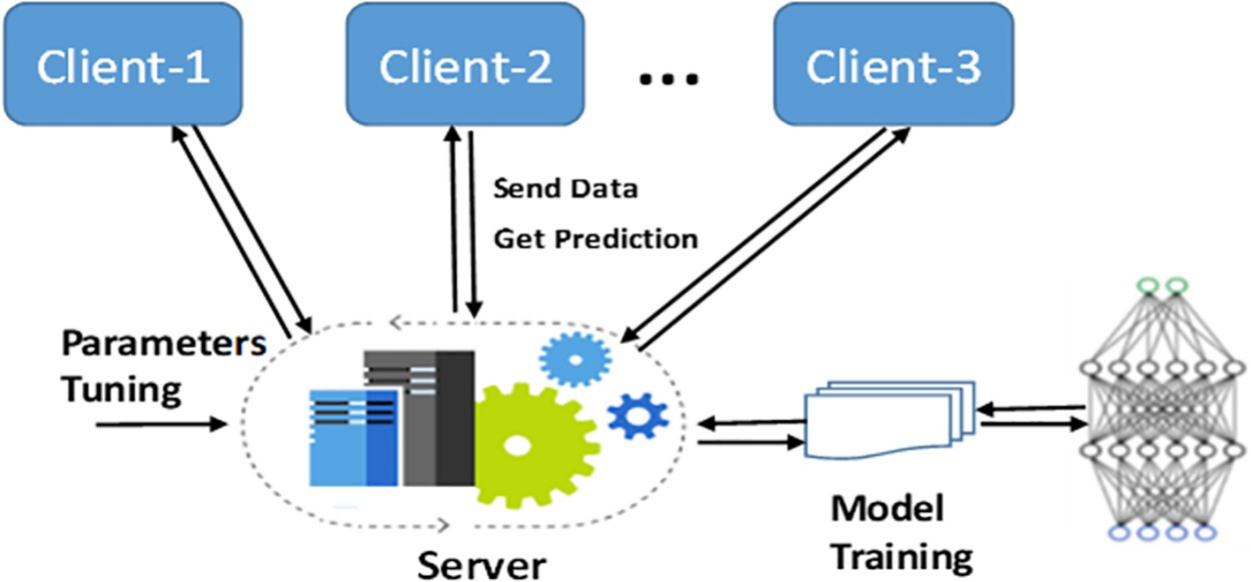
Conventional model training.

In the traditional approach to employing machine learning for decentralizing data, illustrated in Fig 1, there is a centralized technique in which various parties possess the data.

In this setup, every participant sends their data to a central server to train a predictive model. After the training process, the final outcomes are shared with all clients [18]. However, this traditional approach has its pitfalls, mainly regarding data privacy and network dependency. The major issue is the potential for data breaches, as the information must be transmitted from the clients to the central server and can be vulnerable during transmission [19]. Also, to efficiently handle large data and deliver timely predictions, a network connection with high bandwidth and low latency is mandatory. An alternative approach is to use each client’s data to train the ML model and then distribute copies of the trained model to each participant [20–23]. This way, data doesn’t need to be moved when new insights are gained, as each owner has their model. This offers two key benefits over the traditional method. Firstly, the latency problem can be mitigated by making individual predictions for each client. Secondly, it reduces network dependence by cutting communication costs.

### 2.1 Federated learning

**Fig 2:**
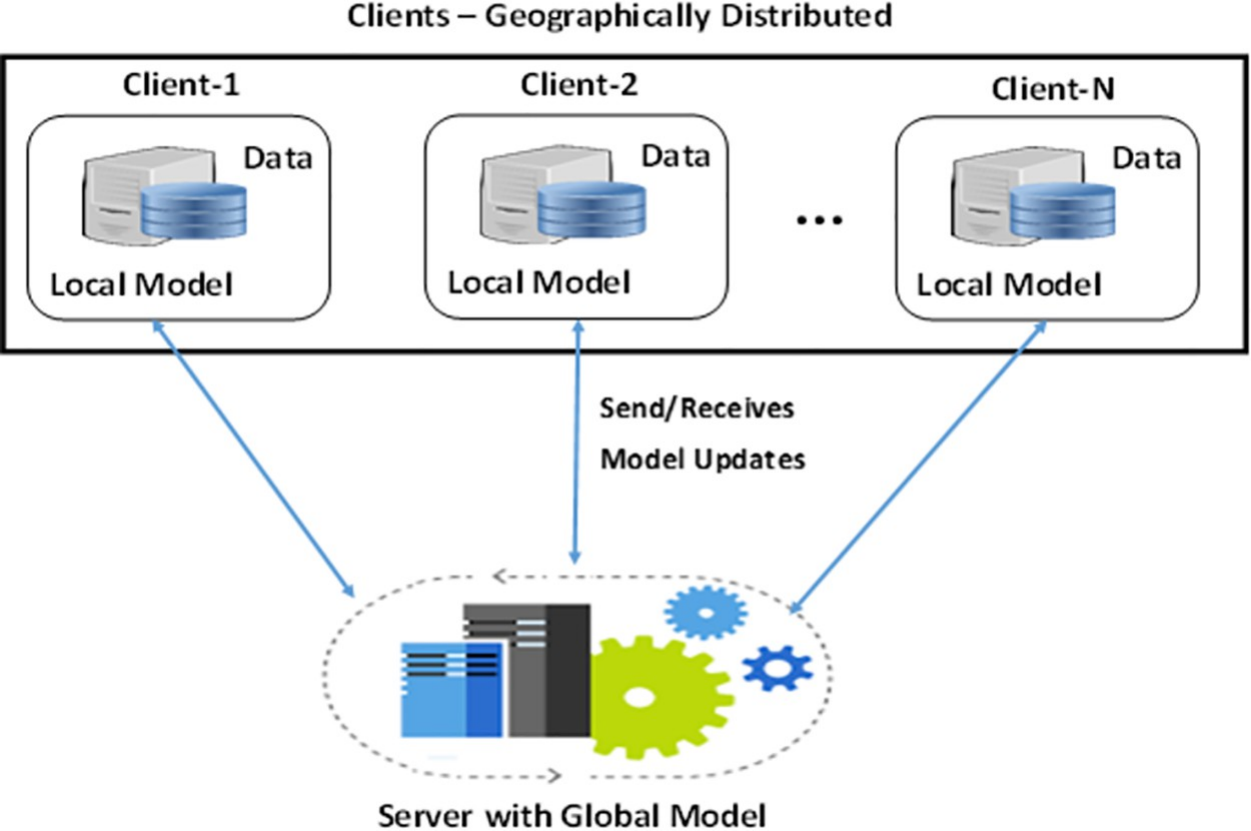
Collaborative model training (Federated learning).

FL is a transformative approach in machine learning, spearheading a new way of learning from distributed data while safeguarding privacy [24]. It permits multiple devices to train a shared model collaboratively, eliminating the need for direct data exchange and addressing significant privacy and security issues. It ensures that data stays on the servers of each data owner, even during training, thus enhancing data privacy, especially when centralized training isn’t feasible due to local data retrieval issues. Instead of anonymizing data, FL assesses information directly at the source, preventing the need to transmit user data to central servers [25, 26]. Consider a scenario involving two hospitals unable to share data, yet both are interested in the same medical data analysis. They must collaborate, using machine or deep learning methods for their analysis and predictions, which a facilitator or server will guide. A real-world example can be seen in medical imaging research. FL can be implemented as follows: Hospital 1’s clients receive model information from the server, use their data to train this model, and send the weights obtained during this training back to the server. The same process occurs with clients in Hospital 2. The server then uses the weights from both hospitals to calculate new aggregated weights. Once these updated weights are ready, they are sent back to the hospitals, where clients reassess their models using these updated weights. This procedure can be repeated as many times as necessary. In the FL framework, the model’s training is executed across distinct entities, termed ‘clients,’ coordinated by a central server. Every client possesses local data for training to update the centrally housed global model. No client data is transmitted to the server; only model updates are shared. FL’s appeal has been growing recently. For instance, the application of FL to smart healthcare informatics is outlined in Fig 2.

In this decentralized and cooperative model, each client uses its own data. After training, each client transmits the weights from the local system to the central server, which aggregates these weights using a specific function to update the global model. The updated model is returned to the clients with these newly aggregated weights. The process is repeated whenever new LMU updates are transmitted to the server. Here are the primary steps of the FL process:

- A Newly created model is posted to the server which is subsequently used by clients for local training.
- Clients initiate training using locally stored data. A portion of the data set is used for testing and performance evaluation of the model.
- Next, the clients send the local model updates to the server transmitting only the local model parameters. This transmission is encrypted to safeguard privacy.
- The model at the server is updated considering the global model weights received from the clients utilizing an aggregation function.

Two types of FL can be distinguished according to data origin: a) Homogeneous FL: here, data from various hospitals with similar attributes are analyzed across all clients. While clients may handle distinct data and samples, they typically share common traits. An example is image classification for detecting different diseases in remote patients. b) Heterogeneous FL: in this scenario, clients can access uniquely featured data with a common identifier. Imagine two hospitals, both clients within the FL framework, each having data from the same users but containing different features. User data can be vertically amalgamated based on the two hospitals’ data. Though this method is more complex and less direct, it’s beneficial in many cross-silo FL scenarios.

FL has a wealthy number of applications. FL’s decentralized nature aligns seamlessly with intelligent big data analysis, which can effectively process and analyze data from various sources without compromising data privacy [27]. FL also has promising applications in renewable energy, particularly optimizing and managing distributed energy resources [28, 29]. Data is generated across diverse geographical locations in renewable energy systems, such as solar or wind farms. FL can be crucial in enhancing the optimization process for DNA protein synthesis [30]. By allowing collaborative training of deep learning models on decentralized, sensitive genetic data, FL ensures privacy and leverages diverse datasets for improved accuracy and robustness in genetic predictions while efficiently handling large-scale genomic data [31].

### 2.2 Federated learning in healthcare

FL holds significant promise in the healthcare industry due to its capacity to facilitate collaborative machine learning without compromising data privacy [32]. Healthcare data is incredibly sensitive and highly regulated, making it challenging to develop robust machine-learning models that can benefit the industry [33]. The traditional method of centralizing patient data from different hospitals or clinics to train a machine-learning model is often not feasible due to privacy concerns and regulatory restrictions. FL solves this problem by allowing data to remain on local devices, like hospital servers, and only sharing the model updates during the learning process. There are several potential applications of federated learning in healthcare [34]. For instance, hospitals worldwide can collaboratively train ML models for disease diagnosis without sharing patient data directly. Each hospital would train the model locally using its own patient data, and only the model parameters would be shared and aggregated to improve the global model. This way, even hospitals with few data samples can contribute to and benefit from the collective knowledge without violating patient privacy.

Additionally, federated learning can support personalized healthcare [35]. For example, wearable devices can collect and use personal health data to train local models for predicting potential health risks. Again, only the model updates, not the sensitive personal data, are shared to refine the global model. However, challenges remain, such as managing data heterogeneity (since every hospital or device may have a different data distribution) and ensuring the robustness and security of the federated learning process [36]. Nevertheless, with continued research and development, federated learning can revolutionize healthcare by enabling global, collaborative learning while respecting data privacy. Additionally, several researchers, including those cited in references [37, 38], proposed specific FL frameworks for detecting COVID-19 through X-rays.

The paper by [39] thoroughly examines FL datasets in healthcare, addressing challenges such as scalability and data quality, but primarily concentrates on privacy and security issues. The study in [33] proposes an FL-based architecture for healthcare informatics, emphasizing the risks of security attacks. However, it does not sufficiently address challenges related to hybrid non-IID features. [40]’s focuses on privacy and data ownership challenges in centralized FL models in healthcare but lacks a practical use case in privacy preservation. The article [41] discusses various FL tools and frameworks, including data collection and preprocessing, but falls short in addressing FL aggregation. In contrast, [42] reviews FL technologies in the biomedical field, highlighting challenges in fragmented healthcare data, yet mainly focuses on pre-trained models and segmentation datasets, overlooking issues arising from data heterogeneity. The research in [35] identifies key factors in designing FL systems for healthcare and evaluates existing frameworks. [36] proposes a CFL application for COVID-19 diagnosis using edge computing, discussing machine learning challenges in privacy-sensitive healthcare contexts but maintains a general approach towards FL. The comprehensive review by [43] on FL in Electronic Health Records (EHR) discusses research issues and potential solutions, yet it lacks an in-depth evaluation of its proposed architecture and does not touch upon ethical concerns related to EHR.

### 2.3 Data heterogeneity in federated learning

Federated Learning (FL) has evolved as a novel approach in machine learning, enabling decentralized training across many devices while upholding user privacy. A key hurdle in FL is the diversity of data, known as data heterogeneity. This term describes the scenario in which the data held by various clients (or nodes) in a distributed network differ in characteristics or are not uniformly distributed. Data heterogeneity in FL encompasses a spectrum of disparities in client data, encompassing differences in data types, formats, quality, availability, and distributions. Such diversity presents substantial challenges in FL, leading to issues such as model performance degradation, convergence problems, and potential privacy violations. For instance, if the data from one client is markedly distinct, it might disproportionately influence the training process, skewing the model toward its data distribution. This could result in subpar data generalization from other clients and diminish the model’s accuracy. In a typical FL environment, various clients (such as devices or servers) contribute their distinct local datasets for model training. The heterogeneity in these datasets stems from differences in:

- Clients in an FL system might possess data from diverse classes or distributions. This variation leads to non-IID (Independent and Identically Distributed) data, where each client’s data set differs significantly in statistical properties.
- The amount of data each client holds can also vary widely. Some clients may have large datasets, while others may have relatively smaller ones.
- The quality of the data, which can be affected by issues like noise or missing values, may not be uniform across different clients. This disparity in data quality can greatly influence the performance and effectiveness of the trained models in an FL system.

To exemplify this concept, imagine an FL system where each client is a distinct hospital. These hospitals have varying patient demographics, disease frequencies, and data collection methods, resulting in highly diverse data. Medical data inherently exhibits variety and complexity, influenced by factors like the origin and format of health records, data dimensionality, and the variety in data acquisition devices and protocols. Current research indicates that this heterogeneity poses challenges for many FL algorithms. An area ripe for future exploration is the creation of FL algorithms specifically designed to handle the complexities associated with heterogeneous data. There has been some advancement in this regard, as highlighted in existing academic works. Data heterogeneity significantly impacts FL, especially in terms of model performance. A globally trained model on such varied data may show effective results on specific clients while underperforming on others, owing to the significant differences in the characteristics of local data.

## 3 Hypothesis and limitations

- **Hypothesis**: Our hypothesis posits that while Federated Learning (FL) offers significant advantages in terms of privacy and decentralized learning, the inherent data heterogeneity in practical scenarios like medical imaging can adversely affect the performance and convergence of FL algorithms. We explore this hypothesis by examining how variations in data distribution impact the efficacy of FL algorithms.
- **Limitations**: Our study, while comprehensive, has limitations. Firstly, our experiments are confined to specific types of datasets and may not encompass all forms of data heterogeneity in medical imaging. Secondly, we focus on the FedAvg algorithm, and our findings may not be generalizedW to all FL algorithms. Lastly, the simulated federated environment, while reflective of real-world scenarios, may not capture all the complexities of actual deployments.

## 4 Material and methods

This research utilizes an experimental methodology to evaluate the impact of data heterogeneity on the performance of FL algorithms. It offers an in-depth understanding of how data heterogeneity affects the convergence and performance of the FL algorithm. It aims to provide insights that could guide the design of more robust algorithms under heterogeneous settings. This section delineates the major steps undertaken in this research, including selecting suitable FL and DL algorithms, dataset selection, data partitioning to simulate varying degrees of heterogeneity, and model training. We used the FedAvg algorithm as it provides an efficient, decentralized approach, allowing us to train on a diverse range of data distributed across multiple devices. This ingenious technique enables us to harness the power of collective intelligence yet ensures individual data never leaves its original device, fostering trust and creating a more inclusive model. We employed CNN and MLP for client local model training due to their unparalleled proficiency in handling image and recognition tasks. CNNs can automatically and adaptively learn spatial hierarchies of features directly from data, eliminating the need for manual feature extraction. This leads to more efficient and accurate model training and, in turn, enhances the quality of our predictions.

The federated data often exhibits a non-IID characteristic. To simulate this environment, we leveraged the TFF framework to generate the required non-IID setting, focusing on the context of data size and sample variation. However, exploring non-IID settings based on features falls outside the scope of our study. Our objective is to investigate and analyze the impact of non-IID scenarios compared to IID, so we also established balanced partitions (IID) to facilitate a comparison of convergence results. Our proposed data partition settings encapsulate two categories: balanced partition (IID) and heterogeneous partition (non-IID). The balanced partition presents a scenario with minimal data heterogeneity. We simulate a more realistic scenario with moderate heterogeneity, where certain devices exhibit imbalanced data distributions. Understanding that this intricate analysis of a client’s data is feasible because we operate within a simulation environment where all data is locally accessible. Due to privacy concerns, inspecting an individual client’s data would be impossible in a federated environment.

### 4.1 Methodology—Procedures

**Fig 3:**
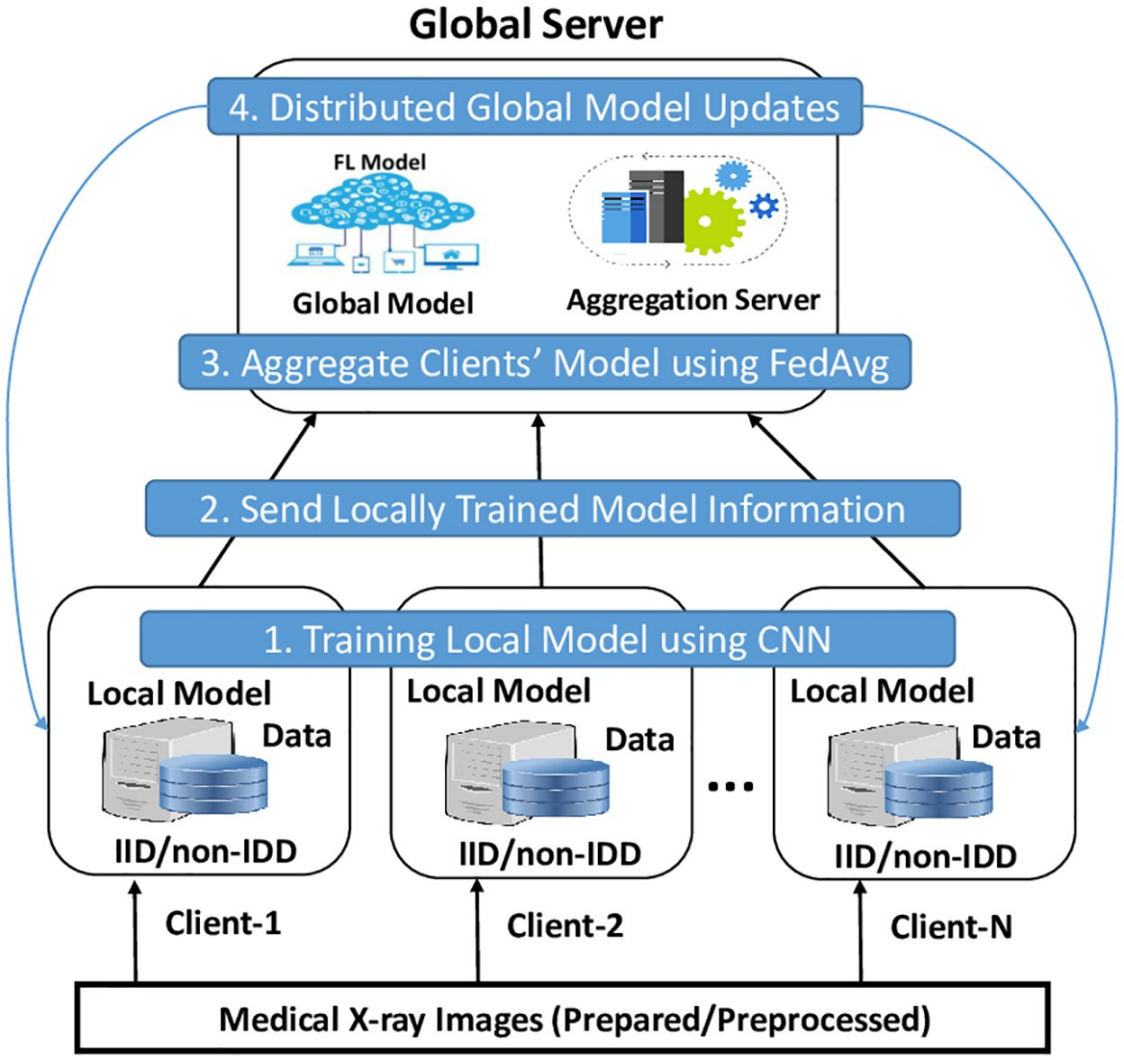
Methodology—Federated learning with different clients.

Fig 3 illustrates the Methodology—Procedure followed in our study. This process can be broken down into the following steps:

- Local Model Training: each participating device uses local data to train a model in FL. This is done using CNN and MLP.
- Sending local model information to the Server: after the local training process is completed, each device sends its locally trained model’s information to a central server. Crucially, the raw data stays on the local device; only the parameters or weights of the trained model are shared, which protects the local data’s privacy.
- Aggregation of Local Model Information: The central server receives the model information from multiple devices. It then uses a technique known as FedAvg to aggregate this information. The server calculates the average of the received model parameters, considering the number of data points used to train each model, to form a global model.
- Distribution of Global Model Updates: Once the global model is formed by aggregating the local model parameters, the server distributes this updated global model back to all participating devices. Each device can then use this updated model as a starting point for the next round of local training, leading to a continuously evolving and improving model.

**Fig 4:**
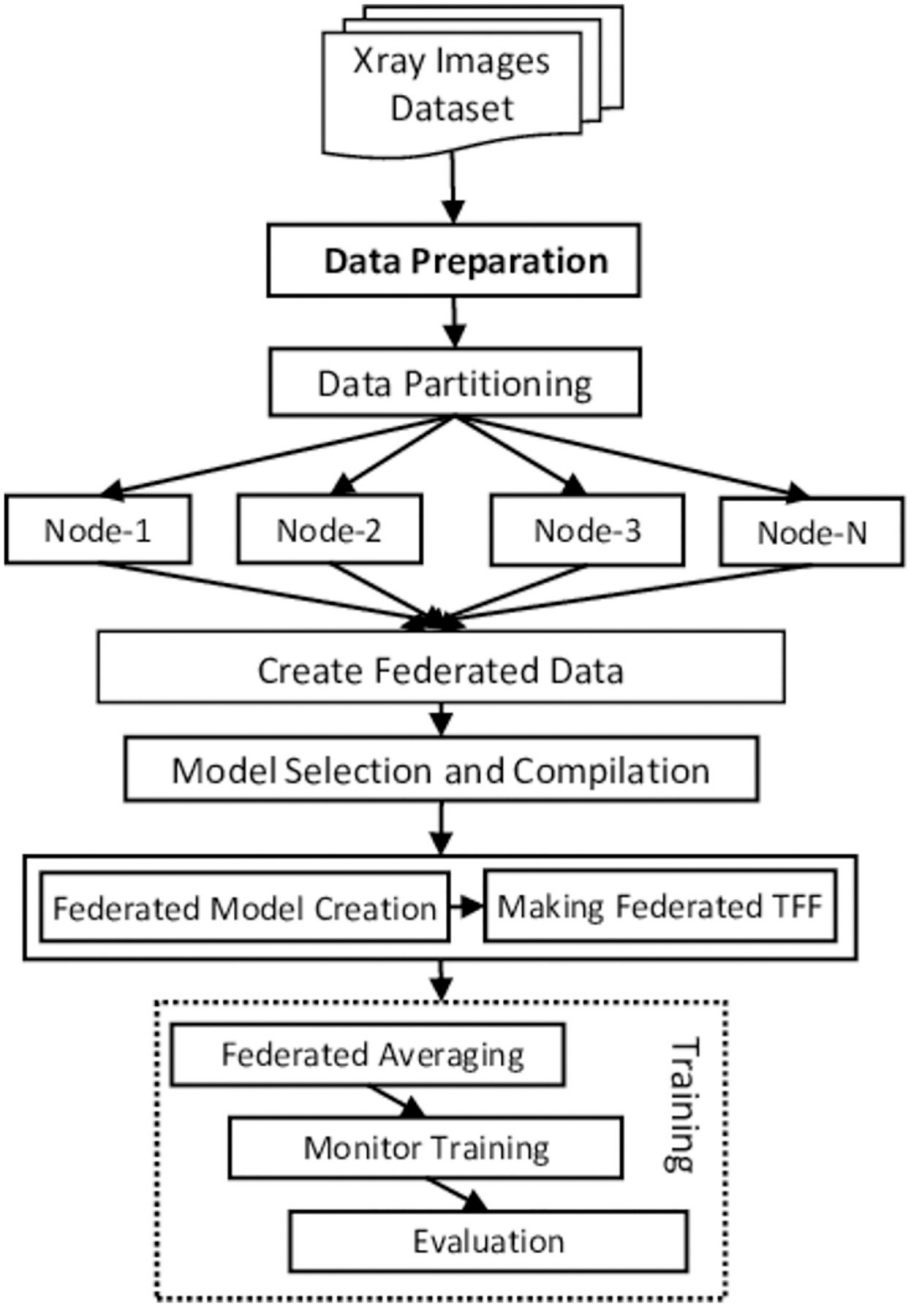
Flowchart.

**Fig 5:**
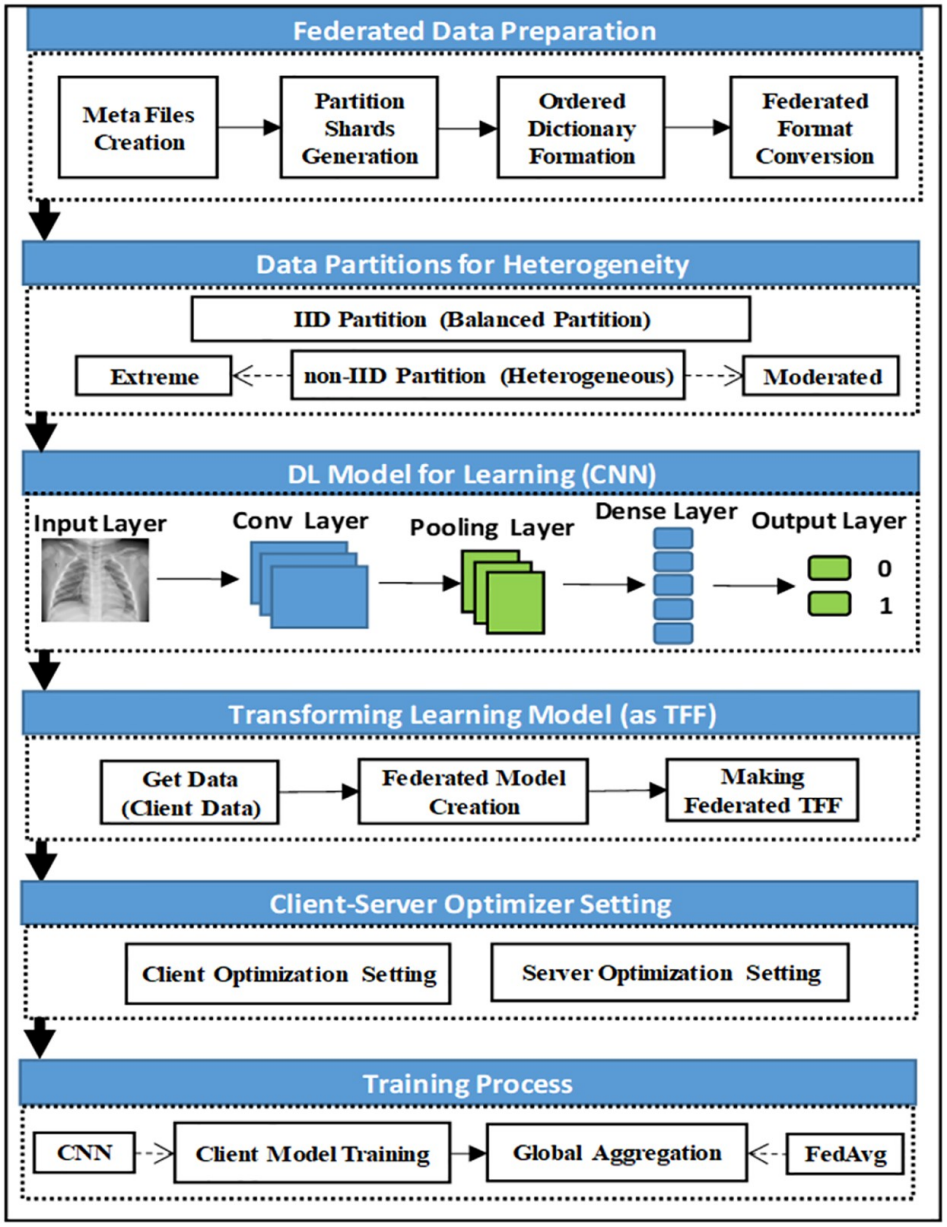
Proposed study—Experimental investigation process.

The workflow of our work is also depicted in Fig 4. Our experiment follows a structured workflow to evaluate FL algorithm performance. Initially, we prepare federated data, distributing it across client devices to simulate an authentic federated environment. We partition data to reflect real-world heterogeneity, and each client uses a CNN to learn from its local subset. The trained CNN models are then transformed into a TFF-compatible format. We set up the optimizer settings for both clients and the server, defining how model parameters will be updated during training. Finally, the FL process involves iterative rounds of local training, model update sharing, server-side aggregation, and distribution of the updated global model. This cycle repeats until we achieve satisfactory model performance or complete a predetermined number of rounds. Through this process, we explore federated learning performance in diverse data scenarios. All the steps are detailed in the subsection of this section. The overall process is also described as Algorithm 1. This algorithm provides a structured approach to conducting federated learning experiments, starting from data preparation to the final output of a trained global model. It covers key aspects such as local model training on each client, server-side aggregation using the Federated Averaging algorithm, and the iterative process of updating and distributing the global model. Additionally, it includes evaluating the model across multiple rounds to assess its performance in terms of accuracy and loss. The proposed experimental investigation process is also shown in Fig 5.

**Algorithm 1** Federated Learning Process for Heterogeneous Data Analysis

**Require**:

1: **Input**: *clients*_*d*_*ata*, *num*_*r*_*ounds*, *num*_*c*_*lients*

2: **Output**: Global model trained across clients

3: **Data Preparation**:

4: Generate meta-file for image classification

5: Organize images into directories by class

6: Create partition shards for training and testing

7: Establish ordered dictionaries for client data mapping

8: Format dataset for federated averaging

9: **Local Model Training**:

10: **for** each client *i* = 1 to *num*_*c*_*lients* **do**

11:  Train local model using CNN or MLP on client *i*’s data

12:  Send model parameters to the server

13: **end for**

14: **Server-side Aggregation**:

15: Initialize global model

16: **for** round *r* = 1 to *num*_*r*_*ounds* **do**

17:  Select a subset of clients for training in round *r*

18:  **for** each selected client *i* **do**

19:   Receive local model parameters from client *i*

20:  **end for**

21:  Aggregate received parameters using FedAvg to update global model

22:  Distribute updated global model to clients

23: **end for**

24: **Model Evaluation**:

25: Initialize lists for tracking training losses and accuracies

26: **for** round *r* = 1 to *num*_*r*_*ounds* **do**

27:  Evaluate global model using federated dataset

28:  Record model’s accuracy and loss

29: **end for**

30: **Final Output**:

31: Return trained global model

32: Return training losses and accuracies

### 4.2 Data preparation and preprocessing

Data readiness and preprocessing are crucial stages in the FL. The importance of data preparation and preprocessing must be considered in the FL workflow. We have executed meta-file generation, organized image directories, created partition shards, established ordered dictionaries, and formatted the dataset for federated averaging. First, we initiated the meta-file generation, creating meta-information that aids in effective data mapping and understanding. Next, we organized the images into well-structured directories to ensure easy access. Understanding the need to optimize data processing and distribution, we divided our data into manageable ‘shards’ by creating partition shards for experiments. Additionally, we established ordered dictionaries to guarantee consistent data retrieval and handling. Lastly, we tailored our dataset structure specifically for the method of federated averaging.

#### 4.2.1 Creation of meta file and directory structure for images

This step is needed to load the image file names and corresponding labels (classes) from the training and testing datasets. Recreating the directory structure of the images is important for organizing image data into separate class directories for data distribution among clients. The directory structure helps to group images by class, making it easier to learn the patterns and characteristics of each class. The images would be organized with a directory structure, and tracking which images belong to which class would be easier. This makes it possible to train models in different data distribution settings. Therefore, by recreating the directory structure of the images and organizing them by class, we can make it easier to prepare and use image datasets for FL. In addition, organizing image data into class directories can help improve ML models’ performance by reducing the likelihood of misclassifying images. When the images are organized by class, the ML model can learn each class’s specific patterns and characteristics more effectively, leading to better accuracy and performance. The above step must create separate directories for each dataset (full, class 1, and class 0) and organize the image data based on their class labels.

#### 4.2.2 Partition shards generation

The pre-arranged directories are read into memory for training or testing. It reads images from three main directories created in the previous step. The directory contains all the data, only COVID-positive and normal images. The data set is loaded in the specified directory path, with the parameters set to infer labels from the directory structure, use a specific batch size, resize images to a specific set of pixels, seed the data shuffling with a predefined constant, and maintain the color channels of the images. It serves several vital functions in the FL workflow. It aids in efficient resource utilization by training the model on image batches instead of loading all images simultaneously, thus preventing memory issues for large datasets. It also practices the standard data separation in FL. Additionally, segregating the different classes into separate datasets allows for analysis of the model’s performance on each class individually, which is particularly useful in cases of imbalanced classes or classes with significantly varying attributes. These steps are crucial in structuring an efficient and thorough FL model development and evaluation process.

#### 4.2.3 Ordered dictionary creation for client mapping

The image dataset is transformed into an ordered dictionary to generate the client mapping process for FL. This process is primarily used in the context of FL to take a TensorFlow object. The goal is to create a set of clients or partitions, where each partition is an ordered dictionary of image data and labels. The function works by iterating through the provided dataset and dividing it into smaller, more manageable batches. Each batch is then assigned to a client. This process repeats until the dataset is fully divided among the clients. This is useful in an FL scenario where the goal is to simulate a distributed environment with multiple clients, each possessing a subset of the total data. For instance, it could mimic how data might be distributed across multiple clients in a real-world FL application. The main reason for creating such a function is to prepare and structure data for FL. In real-world applications of FL, data is typically distributed across many devices, such as different hospitals, where each hospital (client) has its own subset of the total data available. The proposed preprocess step simulates a real-world FL environment by dividing a central dataset into multiple partitions and assigning each partition to a simulated client. By doing this, the function creates a structure mimicking how data is distributed in actual FL scenarios. This partitioning allows the model to be trained decentralized, where each client trains the model on its own subset of the data. The structure also facilitates aggregating the updates from all clients to improve the global model in the FL process.

#### 4.2.4 Formatting dataset for the federated averaging

This step prepares the dataset for the federated averaging algorithm in TFF. This function takes a dataset as an argument, repeats it for a specified number of communication rounds, batches it with a specific batch size, and then applies a mapping process. The mapping process reshapes the pixel data and the labels into a format required by the TFF learning algorithm. The reshaped data is then returned as an ordered dictionary. This preprocessing is essential to ensure the data is in the correct format for the TFF’s FedAvg algorithm. Preparing the data in this way is necessary because TFF has specific requirements for structuring the input data TFF’s FedAvg algorithm expects datasets to be in a specific format, as an ordered dictionary where the keys are the features, and the values are batched features values. The process helps ensure the data is in a compatible format for the FL process. These preprocessing steps facilitate efficient and accurate computations during model training, making it easier for the algorithm to learn patterns from the data.

### 4.3 Data partitions—Simulating data heterogeneity

Two different data partitioning strategies are used to simulate varying degrees of data heterogeneity. (i.) IID partition (balanced): a balanced data partition where each device has an equal number of samples for each class. This represents a scenario with no or minimum data heterogeneity. (ii.) non-IDD (heterogeneous) partition: a non-IID partition where some devices have imbalanced data distributions, representing a more realistic scenario of moderate heterogeneity. In the context of COVID-19 diagnosis using chest X-ray images, the data partitions are considered in the following ways. To create an IID partition, each device is assigned an equal number of total samples, including an equal number of COVID-19 images and non-COVID-19 images from each class, resulting in a balanced data partition where each device has an equal number of samples for each class. In addition, to create a heterogeneous partition, some devices have more COVID-19 images than others due to imbalanced data distributions. To create a moderately heterogeneous partition, some devices can be assigned more COVID-19 images and fewer non-COVID-19 images from each class. In comparison, others can be assigned fewer COVID-19 images and more non-COVID-19 images from each class. This would result in a partition where some devices have imbalanced data distributions, but the overall data distribution is still relatively similar across devices.

This study deliberately focuses on class distribution imbalance for various compelling reasons, presenting a solid justification for our chosen approach. Firstly, addressing class distribution imbalance lays a foundation for broader research endeavors within FL, especially critical during the initial stages of model training and performance assessment. By tackling this issue, we aim to establish a robust base for addressing more intricate challenges, such as feature distribution shifts, thereby enhancing the robustness and efficacy of FL models in medical imaging. The selection of the COVIDx CXR-3 dataset underscores its acute relevance to contemporary global health crises, marked by significant class imbalances mirroring real-world diagnostic challenges for COVID-19. Our investigation into these imbalances seeks to directly refine the accuracy and reliability of COVID-19 detection models within an FL framework, thereby contributing tangible benefits to public health initiatives. Furthermore, focusing on class distribution imbalance offers a scalable and methodologically sound avenue to explore data heterogeneity’s impact on FL, setting a precedent for systematically evaluating imbalance effects on model performance. This approach facilitates a structured examination of current issues and lays a scalable foundation for future research incorporating more complex data variations, such as feature distribution shifts. Our research acknowledges the importance of addressing feature distribution shifts in FL for medical imaging as a vital direction for subsequent studies. Initially concentrating on class distribution imbalance, our work provides a groundwork for expanding into feature distribution shifts, leveraging the insights and methodologies developed to explore broader data heterogeneity aspects in future research.

### 4.4 Network models for federated learning analysis

We used a CNN model, often used in image analysis initially. The CNN model is defined with Keras deep learning framework. The model begins with a reshaped layer to convert the input images to their original 2D form. There are alternating convolutional layers and max pooling layers. The convolutional layers apply a series of filters to the input images to extract features, and the max pooling layers downsample the feature maps, reducing computational complexity and helping with feature invariance. After the convolutional and pooling layers, the feature maps are flattened into a single vector and passed through a dense (fully connected) layer with ReLU activation. A dropout layer is included to help prevent overfitting by randomly setting some of the layer’s inputs to zero during training. The model concludes with a final dense layer that uses softmax activation to output the predicted probabilities for each class. CNN is a type of neural network typically used in image processing tasks due to its exceptional ability to extract hierarchical features from raw input data. Its architecture is inspired by the organization of the animal visual cortex and is particularly effective for tasks involving spatial invariance, where features can be recognized anywhere in the image. In addition, we also utilized the MLP to validate our claim in a strong way. MLP, sometimes called a neural network, is a fundamental type of artificial neural network. It consists of at least three layers of nodes (also known as neurons). These layers include an input layer, one or more hidden layers, and an output layer. MLPs are characterized as a supervised learning technique, meaning they learn from labeled training data to make predictions or classifications. Each node in one layer connects with a certain weight to every node in the following layer.

### 4.5 Transforming the learning model into a TFF model function

Transforming or wrapping the learning model as a TFF model is critical for implementing FL. This step ensures your model is properly encapsulated in a format that TFF can understand and work with. When FL is performed, computations (including model training and evaluation) are distributed across multiple decentralized clients. TFF needs to understand the model architecture, input data specification, loss function, and metrics it will compute. By re-constructing the Keras model as a TFF model function, we ensure that TFF can correctly understand and handle these requirements. This process is used to create a federated dataset for a selected subset of clients, which TFF will use in each round of FL. This function selects a random set of clients for each round of training, providing a way to handle the federated nature of the data and to work with the inherent data heterogeneity in federated learning. This process retrieves the specification of the input data, such as the shape and type of the tensors that your model expects. This input specification ensures that the model and data are compatible and that the training process proceeds without errors. The model is created with the specific input specification and includes a defined metric (Categorical Accuracy). This ensures that the model is compatible with the FL framework. TFF must be aware of the model architecture, the input specifications, and the metrics to track. Its role is to prepare federated data, which means data from different clients or sources. Here, it randomly selects a subset of clients from all available clients. The number of selected clients varies randomly between a quarter and half of all available clients. Then for each selected client, it creates a TensorFlow dataset and applies preprocessing. The result is a list of datasets, one for each selected client, ready for FL. In FL, data from different sources is critical as the core idea is to train a model across different nodes (clients) holding their own local data. It generates a sample dataset from the first client and then applies the same preprocessing that would be applied in training. It then retrieves the specifications from this processed dataset. This specification will guide the model to interpret the incoming data correctly.

### 4.6 Configuring optimizers for simulated TFF client-server learning rates

It defines two major operations, client optimization and server optimization, which specify the optimizer for the client and server, respectively. In this case, the optimizer chosen is Stochastic Gradient Descent (SGD) with a specific learning rate. The optimizer functions are set to control the optimization algorithm and its hyperparameters during the FL process. By specifying the learning rate, we determine the step size at each iteration of the optimization algorithm. A higher learning rate allows for larger updates, which can lead to faster convergence but may also risk overshooting the optimal solution. Conversely, a lower learning rate leads to smaller updates, potentially resulting in slower convergence but with more precision. By customizing the optimizer functions for the client and server separately, it is possible to have different learning rates or even different optimization algorithms, depending on the specific requirements of each component. This flexibility allows for fine-tuning the optimization process to achieve optimal performance in the FL scenario. Setting the optimizer functions for the learning rate in the TFF client-server simulator is vital for controlling the optimization process during FL. Customizing the optimizer functions and learning rates for the client and server components allows us to control the optimization process in FL. It enables us to address data heterogeneity, control the impact of individual clients on the global model, and optimize the convergence speed and stability of the learning process.

- Client-Side Optimization: The client optimizer function determines how the local models on the individual clients are trained. By specifying the optimizer with a particular learning rate, we can control how quickly the client models converge and how they adapt to the local data. Different clients may have different data distributions, and setting individual learning rates can help account for this heterogeneity.
- Server-Side Aggregation: The server optimizer functions for the aggregation of client models and the updates to the global model. During federated averaging, the server aggregates the model updates received from multiple clients to generate a new global model. The server optimizer with a specific learning rate allows us to adjust the weight given to each client’s update during aggregation. By modifying the learning rate, we can control how the global model incorporates information from different clients.
- Optimization Control: We can fine-tune the balance between local adaptation and global aggregation by setting different learning rates for the client and server optimizers. This control helps ensure effective client collaboration while maintaining convergence and stability during the federated learning process.

### 4.7 Server-side model aggregation (Global model training)

Model aggregation is a process used in FL to combine the model updates from different clients into a single global model. The goal of model aggregation is to ensure that the resulting model is accurate and representative of the underlying data distribution across all clients while accounting for differences in data distribution and ensuring that the training process is fair. In FL, model aggregation can be performed using various methods, such as simple averaging, weighted averaging, and federated averaging. Simple averaging involves taking the average of the model updates from all clients and using it as the new global model. Weighted averaging involves assigning weights to the model updates based on the importance of each client’s data to the overall data distribution. Federated averaging is a more advanced method that uses a weighted average of the model updates, where the weights are determined using a combination of local model accuracy and the amount of data held by each client. This study uses the FedAvg algorithm for each data partition. Every device computes a local model update during each communication round using its data and sends it to the server. The server then averages these updates to create a global model update. This global model is then returned to the devices for the next round of updates. The process repeats until convergence or a predetermined number of communication rounds. This process involves training a global model using data from multiple clients in a distributed manner. First, the function initializes empty lists to store the training losses and accuracies. These metrics will be recorded for each round of training. The input specification for the federated data is retrieved, which helps ensure compatibility between the model and the data. Next, the federated averaging algorithm is constructed using the FedAvg() function. This algorithm handles the aggregation of client model updates and updates the global model. The client and server optimizers are specified within the algorithm. The training process then proceeds in a loop for the specified rounds. Each round creates a federated dataset by randomly selecting a subset of clients’ data. The federated averaging algorithm performs one round of training using the selected dataset, updating the server state. The accuracy metric is extracted from the training results during training in the proposed study. The accuracy values for each communication round are stored in the respective lists. The current round’s accuracy is recorded for monitoring purposes. This training process uses federated averaging, allowing for collaborative training across multiple clients while aggregating their model updates to obtain a global model. The FedAvg algorithm is described as Algorithm 2.

**Algorithm 2** Training Process using Federated Averaging

**Require**:

1: **Input**:

2: *clients*_*data*: Federated dataset containing data from multiple clients.

3: *num*_*rounds*: Number of training rounds.

4: Initialize empty lists: *train*_*losses*, *train*_*accuracy*

5: Retrieve input specification using the *get*_*input*_*spec* function.

6: Create federated averaging algorithm using the *tff*.*learning*.*algorithms*.*build*_*weighted*_*fed*_*avg* function, with the model function, client optimizer function, and server optimizer function.

7: Initialize the federated averaging algorithm to get the initial server state.

8: **for** *round*_*num* in *range*(*num*_*rounds*) **do**

9:  Create a federated dataset using *make*_*federated*_*tff*_*data* by randomly selecting a subset of clients’ data for this round.

10:  Perform one round of training using *federated*_*averaging*.*next* and update the server state.

11:  Get the model weights using *federated*_*averaging*.*get*_*model*_*weights*.

12:  Extract training metrics such as accuracy and loss from the result.

13:  Append the accuracy value to *train*_*accuracy*.

14:  Print the current round’s accuracy.

15: **end for**

16: Clear the TensorFlow session using *K*.*clear*_*session*().

17: **return** *train*_*losses* and *train*_*accuracy*

In our study, we embark on a novel journey, customizing the FedAvg algorithm, traditionally utilized across various Federated Learning (FL) scenarios, to specifically address the challenges posed by medical imaging datasets. This customization involves adjusting the algorithm’s parameters and aggregation methods to suit the complex data structures and distribution patterns of medical images, focusing on the COVIDx CXR-3 dataset. We provide a comprehensive discussion on the FL process and the specifics of FedAvg implementation in Section 2.1 of our manuscript, ensuring that our approach is tailored to medical imaging data. Additionally, our experimental setup involves creating an FL environment with multiple clients and distributing the COVIDx CXR-3 dataset among them in both IID and non-IID configurations. This is crucial for assessing the algorithm’s performance across different data distribution scenarios. Uniquely, our research pioneers the application of the FedAvg algorithm to the COVIDx CXR-3 dataset, exploring the performance implications of IID versus non-IID data setups, marking a novel approach in the FL field, especially in the context of the current pandemic, as opposed to the more commonly studied datasets in FL research.

## 5 Results and discussion

### 5.1 Implementation detail

- Google Collab is used for implementation and experiments with a RAM of 12 GB and a disk space of 358 GB. Also, the Google Collab Pro version is utilized for some of the high computation required tasks, providing more RAM and more time for run-time.
- Kaggle API is utilized to load the dataset to Google Collab and use it.
- To simulate this environment, we leveraged the TensorFlow Federated (TFF) framework to generate a non-IID and IID setting. TFF is utilized to perform experiments on and implement the federated learning algorithms.
- Python programming language is used with Jupiter Notebook.

### 5.2 Datasets

The study employs the COVIDx CXR-3 dataset as the major one and two other datasets for the medical imaging task, as shown in Table 2. Three different X-ray image datasets were utilized in our study. These datasets comprise a vast collection of chest X-ray images designed explicitly for COVID-19 detection research. It is highly relevant to medical imaging and provides a suitable testbed for our study due to the inherent heterogeneity in medical images arising from differences in patient demographics, disease stages, and imaging techniques.

- Dataset-I: This dataset consists of 1,726 X-ray images with a size of 2GB. Among these images, 460 were categorized as positive, and the remaining 1,266 were classified as normal.
- Dataset-II: With a total size of 4GB, this dataset comprises 9,544 X-ray images. Among them, 5,500 images were labeled positive, while 4,044 were identified as normal.
- Dataset-III: This is the largest dataset we utilized in our study, with a hefty size 14GB. It consists of 29,986 X-ray images, of which 15,994 were designated positive and the remaining 13,992 were normal.

**Table 2:** Datasets detail.

Dataset	Size	Total Images	Positive	Normal	Description	Ref
Dataset-I	2GB	1726	460	1266	X-ray images	[44]
Dataset-II	4GB	9,544	5500	4044	X-ray images	[45]
Dataset-III	14GB	29,986	15994	13992	X-ray images	[46]

Our decision to focus on medical imaging, particularly chest X-rays, stems from several vital considerations unique to the healthcare sector that make it an ideal domain for exploring federated learning (FL) applications. The primary rationale is the importance of data privacy and security in healthcare. Medical datasets contain susceptible information, necessitating stringent privacy protections that FL is uniquely positioned to address. This concern is less pronounced in domains such as autonomous driving or IoT, where data privacy, while still necessary, does not carry the same level of personal sensitivity. Moreover, the healthcare sector faces significant challenges related to data heterogeneity, not only in distribution but also in the variability of imaging equipment, protocols, and patient demographics across institutions. This heterogeneity poses unique challenges for FL, as algorithms must be robust to data quality and representation variations. Addressing these challenges within the medical imaging domain can lead to significant advancements in FL, potentially improving diagnosis accuracy and patient outcomes across diverse clinical environments.

Additionally, the potential impact of improving FL in medical imaging is profound. Enhancements in this area could directly contribute to better healthcare delivery by enabling the development of more accurate and privacy-preserving diagnostic tools. This focus allows our research to contribute meaningful insights and advancements that can immediately benefit patient care. Given the unique privacy concerns, data heterogeneity, and potential for high-impact outcomes in healthcare, we believe that medical imaging presents an especially compelling case for FL research. While other domains certainly face data heterogeneity, combining these factors in healthcare underscores the importance and urgency of addressing these challenges in medical imaging first and foremost. Our study thus aims to provide valuable contributions to the FL community, with implications that extend beyond the healthcare domain. We acknowledge the vast potential for FL across various fields and the importance of diversifying research efforts. However, given the scope and resources of a single study, focusing on a comprehensive, high-impact area such as medical imaging allows us to explore deeply the nuances and challenges of FL in a context where its application can be truly transformative.

In our research article, we thoroughly analyzed the behavior of federated learning algorithms on diverse datasets, mainly focusing on medical imaging. Our experiments were specifically designed to understand the impact of data heterogeneity on the convergence of these algorithms. By analyzing these diverse datasets, we ensured the robustness and validity of our study. This multi-dataset approach provided us with a comprehensive understanding of the behavior of federated learning algorithms in heterogeneous data environments, adding depth and dimension to our findings. The datasets’ variety in size, complexity, and composition allowed us to stress-test the limits of federated learning algorithm performance, specifically in model convergence. Our findings thus contribute significantly to the ongoing discussions about the efficacy and potential improvements in federated learning algorithm design and implementation.

### 5.3 Experimental results

We have performed several experiments by varying the number of clients collaborating in the learning process, communications rounds, and deep learning models.

#### 5.3.1 Experiment 1

**Fig 6:**
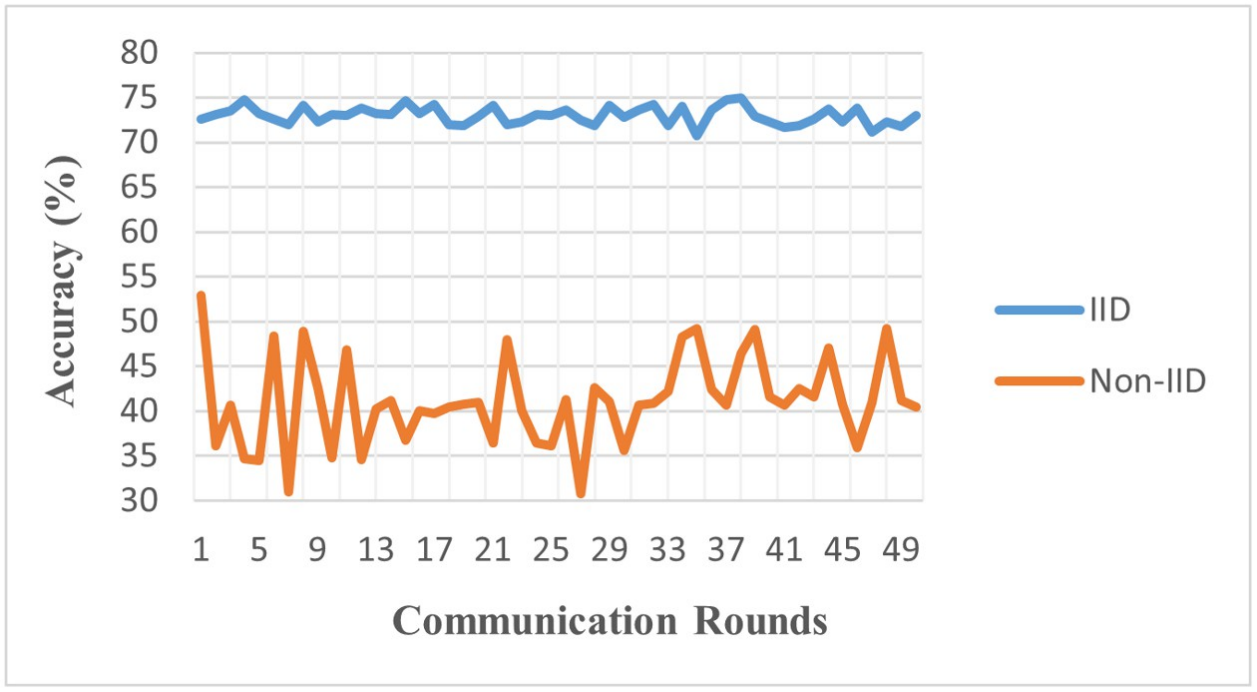
Accuracy of IID vs. non-IID (Model: CNN, Clients: 10, Rounds: 50).

Experiment 1 simulated a scenario involving 10 clients and 50 communication rounds using Dataset-I [34]. Table 3 summarizes the global accuracy achieved with different data partitions using Dataset-I. Our setup utilized the Federated Averaging algorithm (FedAvg) and a CNN as the deep learning model. The experiment was designed to probe the effects of data heterogeneity. We observe that even with the superior model, the issue of data heterogeneity and its impact on convergence in the FL scenario persists. In an IID data environment, where data points share homogeneity, a global accuracy of 0.731, or 73.1%, was achieved, suggesting the model performed well in that context. However, the results differed in a non-IID or heterogeneous data environment. In this setup, where data across clients can be vastly different, the global accuracy dropped significantly to 0.411 or 41.1%. This decrease in performance underscores the enduring challenge that data heterogeneity presents to the effective convergence of federated learning algorithms. The Fig 6 demonstrates the results of experiment 1. We note that this decrease in the performance of non-IID settings is because of the data skew, class imbalance, and feature distribution mismatch. The non-IID setup is simulated unevenly distributed among clients, creating a phenomenon known as data skew. In non-IID settings, some clients have more samples compared to others making it difficult for the global model to generalize well. Another setup is a class imbalance, where individual clients are equipped with large amounts of data. This can negatively impact the global model’s performance, particularly on underrepresented classes. It is also evident from the results that the IID setup results are converging, and much fluctuation can be observed in the global accuracy On the other hand, the non-IID accuracy fluctuates and has performance convergence problems as well.

**Table 3:** Global accuracy using CNN.

Clients	Rounds	FL Algorithm	Model	Data Partitions	Accuracy
10	50	FedAvg	CNN	IID Environment	73.1%
				non-IID Environment	41.1%

The notable disparity in global accuracy between the IID (73.1%) and non-IID (41.1%) environments using the CNN model on Dataset-I starkly illustrates the challenges posed by data heterogeneity in FL. This significant drop in performance in non-IID settings underscores the critical need for FL algorithms resilient to diverse data distributions, particularly in medical imaging contexts.

#### 5.3.2 Experiment 2

In this experiment, we observe the performance of the FedAvg algorithm and MLP model in diverse circumstances with 10 users and 50 communication rounds. In the IID environment, where all data points have the same probability of being observed and are independent of each other, the global accuracy achieved was 73.8% suggesting that the federated learning approach with the FedAvg algorithm and MLP model can perform well in an IID environment. On the other hand, as we tested in the non-IID environment, the assumption of identical distribution and independence does not hold, the global accuracy dropped significantly to 56.2%. This lower accuracy indicates that our model and algorithm faced challenges adapting to the heterogeneity and dependencies in a non-IID environment. These results highlight the potential influences of data distribution and the environment on the effectiveness of federated learning approaches. Further investigation would be necessary to enhance the model’s performance in non-IID environments. The results from this experiment can be seen in Table 4.

**Table 4:** Global accuracy using MLP.

Clients	Rounds	FL Algorithm	Model	Data Partitions	Accuracy
10	50	FedAvg	MLP	IID Environment	73.8%
				non-IID Environment	56.2%

**Fig 7:**
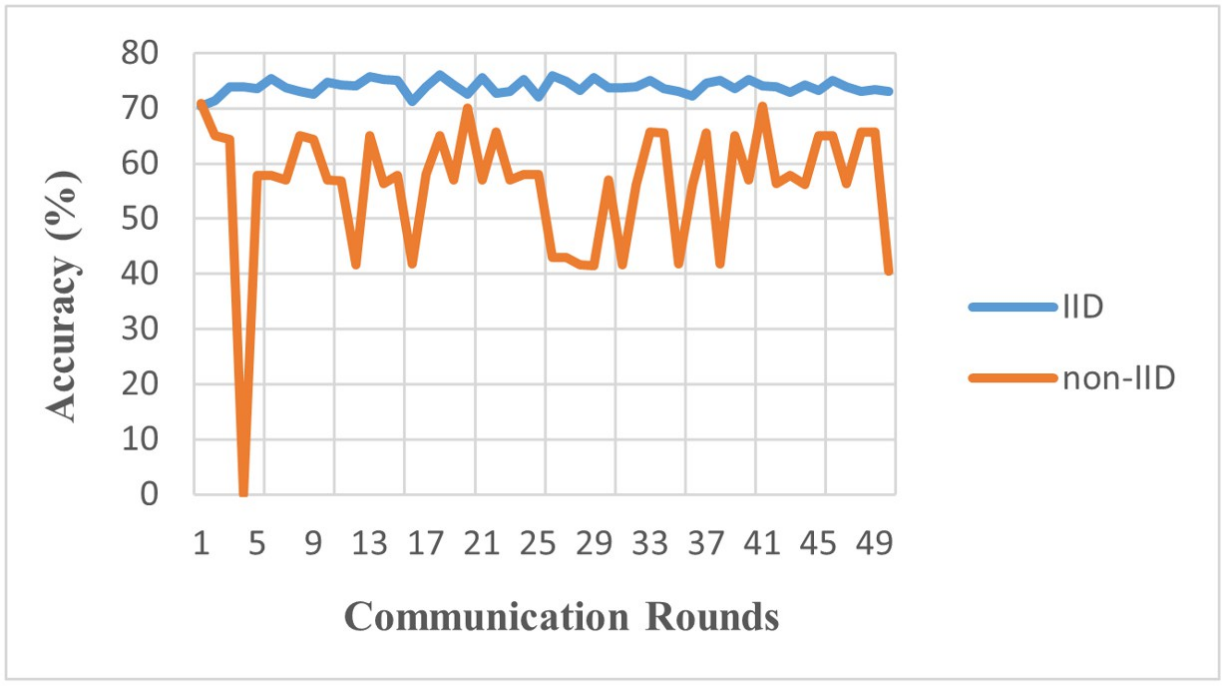
Accuracy of IID vs. non-IID (Model: MLP, Clients: 10, Rounds: 50).

Fig 7, showcases the comparison between the IID and non-IID environments. When the data is IID, each point is generated independently from the same distribution. Under such conditions, each client’s local dataset in a federated learning setup represents a fair sample of the overall data distribution. This allows the model to learn a comprehensive and balanced view of the data, aiding in the convergence of the global model, as seen in the faster and steadier progress towards convergence in the IID environment graph. On the other hand, in non-IID environments, the data distribution varies across clients. This may reflect the real-world situation more closely, where each client (like a mobile device or a user) may have a different data distribution. This discrepancy in local data distributions can pose challenges for federated learning, as it’s harder to generalize a global model from these diverse local models. This is evident from the slower and more fluctuating convergence pattern observed in the non-IID environment graph. This difference between the two environments underscores the impact of data distribution on the effectiveness of federated learning. We observe while federated learning can indeed work under non-IID conditions, achieving stable and rapid convergence is more challenging.

The results from Table 4, highlighting the performance in IID (73.8%) and non-IID (56.2%) environments using the MLP model, demonstrate the complexities introduced by data heterogeneity. The considerable decline in performance in non-IID settings indicates that while MLP models can adapt to homogenous data distributions, their efficiency is markedly reduced in the face of heterogeneous data. This suggests a need for adaptive learning strategies within MLP frameworks for better handling data variability in medical imaging.

#### 5.3.3 Experiment 3

Table 5 outlines the results from Experiment 3, where an FL setup was employed with 20 clients and 50 rounds of communication. In the IID Environment, the global accuracy achieved was 0.729 or 72.9%. This indicates that the global model, trained collectively with data from all clients, could correctly predict the output in approximately 72.9% of the cases. In the non-IID Environment, where the data distribution varies across clients, the global accuracy achieved was 0.576 or 57.6%. From these results, it is clear that the data distribution plays a significant role in the performance of federated learning systems even if the number of clients have increased. With 20 clients, the proposed system performed better in an IID environment than IID to a non-IID environment.

**Table 5:** Global accuracy with more clients.

Clients	Rounds	FL Algorithm	Model	Data Partitions	Accuracy
20	50	FedAvg	MLP	IID Environment	72.9%
				non-IID Environment	57.6%

**Fig 8:**
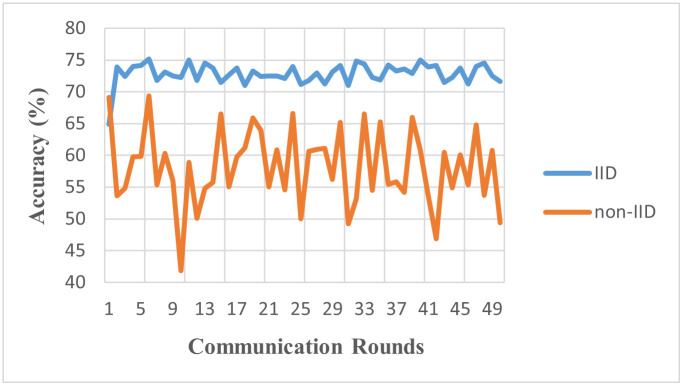
Accuracy of IID vs. non-IID (Model: MLP, Clients 20, Rounds: 50).

Fig 8 vividly illustrates the evolution of global accuracy of experiment 3 across each communication round in the federated learning process. Even as the number of clients participating in the learning increases, a compelling observation emerges: the issue of data heterogeneity continues to impact the model’s convergence. Indeed, an increase in the number of clients introduces a broader spectrum of data representations, potentially enriching the global model’s understanding. However, this expansion is not without its challenges. As this wealth of data is infused into the learning process, the diversity or heterogeneity within the data continues to exert its influence, affecting the model’s ability to converge effectively. In other words, while increasing the number of clients expands the collective learning scope, it also intensifies the existing challenge of data heterogeneity in the federated learning landscape.

Table 5 showcases the impact of increasing the number of clients in FL. While the IID environment yielded a global accuracy of 72.9%, the accuracy dropped to 57.6% in a non-IID setting with 20 clients. This suggests that an increase in client numbers exacerbates the challenges posed by data heterogeneity. More clients introduce a wider array of data characteristics, demanding more robust and adaptable FL algorithms to maintain performance consistency across diverse datasets.

#### 5.3.4 Experiment 4

**Fig 9:**
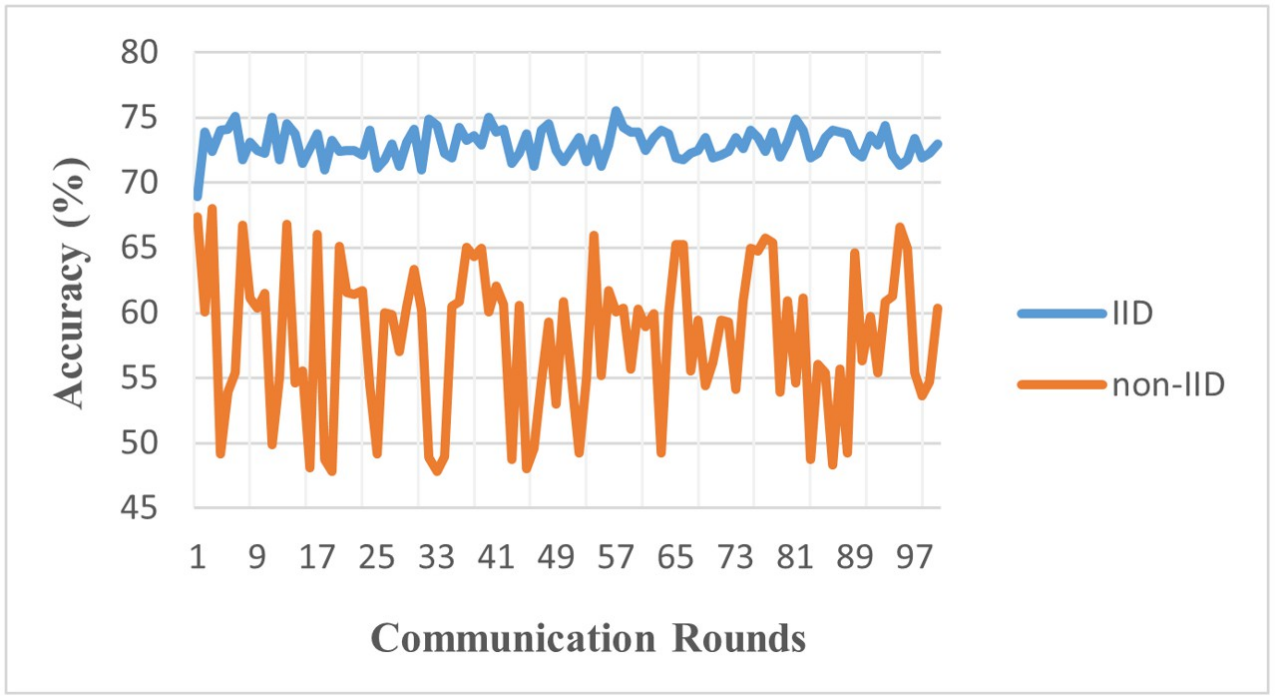
Accuracy of IID vs. non-IID (Model: MLP, Clients 20, Rounds: 100).

Table 6 presents the findings of Experiment 3, a scenario involving 20 clients with an increase in communication rounds to 100. A global accuracy of 0.731 or 73.1% for the IID environment was achieved. On the contrary, the non-IID environment, characterized by data heterogeneity, achieved a global accuracy of 0.580 or 58%. This lower accuracy underlines heterogeneity’s persistent challenges to federated learning, even with increased communication rounds. The central focus of Experiment 3 was to explore whether boosting the communication rounds between client and server could mitigate the convergence issue often seen in a heterogeneous or non-IID environment. Despite the increased number of interactions, the results emphasize the enduring nature of the problem. Even with more opportunities for the server to learn from the clients, data heterogeneity impedes the smooth convergence of the federated model. The accuracy and model loss against each communication round is shown in Fig 9. In this experiment, the model developed using non-IID data tends to adapt too closely to the specific data distribution of their local environment, making them less effective at generalizing to global data distribution.

**Table 6:** Global accuracy with more communiation rounds.

Clients	Rounds	FL Algorithm	Model	Data Partitions	Accuracy
20	100	FedAvg	MLP	IID Environment	73.1%
				non-IID Environment	58.0%

As depicted in Table 6, extending communication rounds to 100 in a setup with 20 clients resulted in slight improvements in global accuracy in both IID (73.1%) and non-IID (58.0%) environments. The persistent gap in performance between IID and non-IID settings, even with increased communication, underscores the complexity of achieving convergence in heterogeneous data scenarios. This finding suggests that while increasing communication rounds may aid convergence, addressing the core challenges of data heterogeneity requires more intricate solutions.

#### 5.3.5 Experiment 5

**Fig 10:**
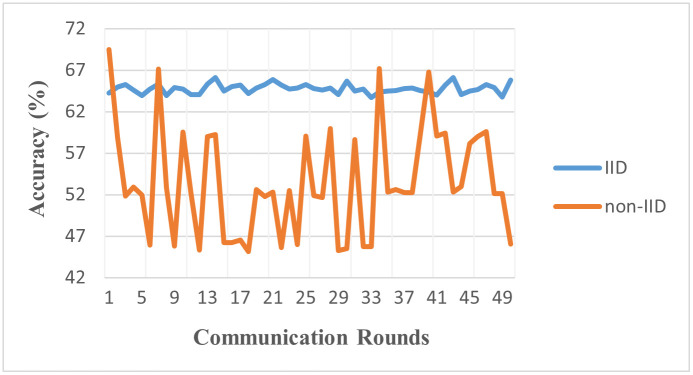
Accuracy of IID vs. non-IID using Dataset-II (Model: MLP, Clients: 10, Rounds: 50).

To further delve into the convergence issue in FL, we utilize Dataset-II [35], a notably larger data corpus. As per the parameters in Table 7 presents the results for the experiment conducted with 10 clients, for 50 communication rounds. In the IID data environment, characterized by homogeneity among data points, we achieved a global accuracy of 0.647, or 64.7%. the global accuracy dropped to 0.534, or 53.4%. Fig 10 illuminates the negative influence of heterogeneity on the performance of the FL algorithm.

**Table 7:** Global accuracy using dataset-II.

Clients	Rounds	FL Algorithm	Model	Data Partitions	Accuracy
10	50	FedAvg	MLP	IID Environment	64.7%
				non-IID Environment	53.4%

Despite using a larger dataset, the challenge of achieving convergence in a non-IID or heterogeneous environment remained apparent. Thus, experiment 5 provides us with a crucial insight emphasizing the need for continued exploration and development of techniques to improve the convergence of FL algorithms, particularly in environments with non-identically distributed data.

The use of Dataset-II in Table 7’s results reflects a similar trend, where the global accuracy in IID environments (64.7%) outperforms that in non-IID environments (53.4%). The results reinforce the notion that as datasets increase in size and complexity, the challenges posed by non-IID data become more pronounced, affecting the efficacy of FL models. This emphasizes the necessity for scalable and flexible FL approaches that can adapt to varying data sizes and distributions.

#### 5.3.6 Experiment 6

In the final experiment, we worked with Dataset-III with a size of 14GB. This dataset encompasses 29,986 X-ray images, 15,994 images were used for training while 13,992 were used for testing. The parameters of this experiment, as shown in Table 8, involved 10 clients, each engaging in 25 rounds of communication. In an IID environment where the data is homogeneously distributed, we achieved a global accuracy of 0.608, or 60.8%. Upon transitioning to a non-IID environment characterized by data heterogeneity, the global accuracy decreased to 0.549, or 54.9%.

**Table 8:** Global accuracy using Dataset-III.

Clients	Rounds	FL Algorithm	Model	Data Partitions	Accuracy
10	20	FedAvg	MLP	IID Environment	60.8%
				non-IID Environment	54.9%

**Fig 11:**
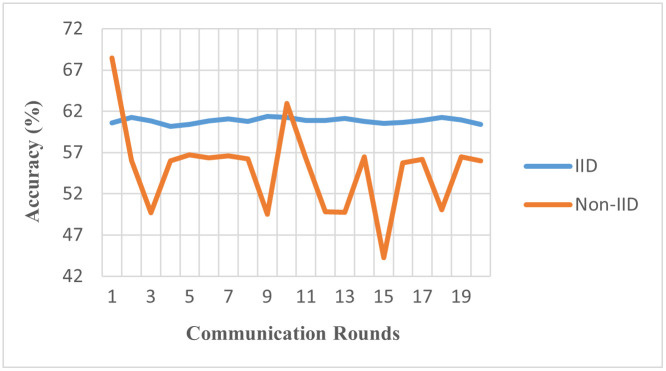
Accuracy of IID vs. non-IID using Dataset-III (Model: MLP, Clients: 10, Rounds: 20).

We observe that utilizing a significantly larger dataset did not alleviate the problem with model performance in federated learning in data heterogeneity. It becomes increasingly clear that the issue of achieving convergence in federated learning algorithms when confronted with non-identically distributed data is a persistent and significant challenge Fig 11 represents these results, illuminating the negative influence of heterogeneity on the performance of the FL algorithm.

In Table 8, employing the largest dataset, Dataset-III, we observe a global accuracy of 60.8% in IID settings and a reduced 54.9% in non-IID scenarios. This highlights a critical observation: even with extensive datasets, non-IID data significantly impacts the performance of FL models. The reduced performance gap compared to smaller datasets suggests that larger datasets may provide a broader representation, aiding model generalization. However, the challenge of data heterogeneity remains significant and needs to be addressed for optimal FL application in large-scale medical imaging

### 5.4 Discussion on results and method evaluation

#### 5.4.1 Analysis of results

Results from the experimental study have highlighted key observations on the impact of data heterogeneity on the performance and convergence of federated learning algorithms, in particular, FedAvg. Results indicate that federated learning algorithms struggled with convergence in non-IID or heterogeneous environments across a series of experiments with varying parameters and across three distinct datasets of different sizes and compositions. The challenge persisted regardless of the change in the number of communication rounds between client and server, increase in number of clients, or use of different models such as MLP and CNN.

**Fig 12:**
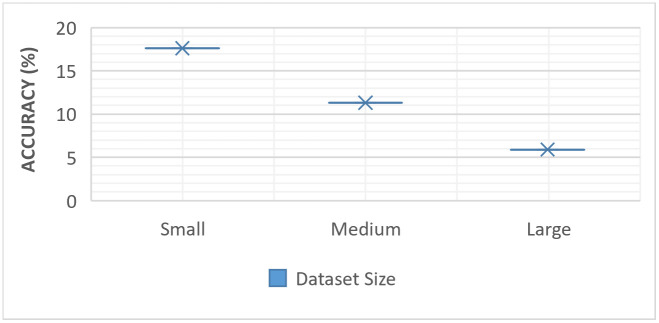
Performance decrease comparison with respect to dataset size.

Our experiments shed crucial light on the impact of data heterogeneity in the context of dataset size, as shown in Fig 12. It is evident that non-IID setups significantly degrade global accuracy compared to their IID counterparts utilizing three different datasets that are Small (2GB), Medium (4GB), and Large (14GB). The degradation in performance varies according to the size of the dataset, with the Small dataset experiencing a notable 17.6% drop in accuracy, the Medium dataset registering an 11.3% decline, and the Large dataset showing a 5.9% decrease. Interestingly, the magnitude of the decrease in accuracy lessens as the dataset size increases. This can be attributed to clients having more data samples and therefore achieving greater convergence. When clients are rich in data samples, there is a reduced likelihood of diminished accuracy. This suggests that the challenges of data heterogeneity become somewhat less pronounced as the dataset size increases, but they remain a pertinent issue to address for optimizing FL algorithms.

These observations underscore that data heterogeneity can significantly impede the effectiveness of federated learning. Our study, therefore, emphasizes the need to develop and integrate strategies or mechanisms that can mitigate the negative impacts of data heterogeneity on FL convergence. This will enhance the robustness of federated learning in medical imaging and pave the way for better and more reliable deployment of these algorithms in healthcare and beyond.

#### 5.4.2 Salient features of the proposed study

- Enhanced Data Privacy: As evidenced in the experiments, our federated learning (FL) approach excels in maintaining data privacy. Processing data locally at the client level and only sharing model updates significantly reduces the risk of sensitive data breaches. This aspect is particularly advantageous in medical imaging, where patient confidentiality is paramount. The method’s decentralized nature ensures that no single point of failure exists, enhancing data security across the network.
- Efficient Utilization of Decentralized Data Sources: The experiments demonstrate the method’s proficiency in leveraging decentralized data sources. This is particularly useful in medical settings where data is scattered across various institutions. Our method effectively harnesses this distributed data, leading to more comprehensive and diverse learning models.
- Reduced Bandwidth Requirements: As observed in the various experimental setups, the FL approach significantly reduces bandwidth requirements by transmitting only model updates rather than the entire dataset. This makes it feasible for environments with limited network resources.
- Impact of Data Heterogeneity: A critical challenge, highlighted across all experiments, is the method’s sensitivity to data heterogeneity. Non-IID data environments consistently resulted in lower accuracy, indicating a struggle to handle diverse data distributions effectively. This issue is particularly pronounced in real-world healthcare scenarios where data variability is inherent.
- Dependence on Client Participation and Quality: The method’s effectiveness is contingent on active and quality client participation. In scenarios like Experiments 3 and 4, where the number of clients increased, the convergence issues became more complex, suggesting a challenge in managing diverse client contributions.
- Scalability Concerns: While the method shows promise, scalability remains a concern, especially when dealing with large datasets and numerous clients. As seen in Experiment 6 with Dataset-III, managing extensive data in a federated setup without compromising performance is challenging.

The federated learning approach presents a paradigm shift in handling medical imaging data, offering significant privacy and decentralized learning benefits. However, the experiments reveal a consistent struggle with data heterogeneity, a critical hurdle in practical healthcare applications. While the approach shows potential, especially in scenarios with limited data diversity (IID environments), its application in real-world, varied medical datasets requires further refinement. The future focus should be on developing strategies to mitigate the challenges posed by non-IID data. This could involve advanced aggregation methods, personalized learning models, or adaptive algorithms that can better handle the diversity inherent in medical datasets. Additionally, scalability solutions need to be explored to ensure that the method remains effective as the number and size of datasets grow. While the federated learning method shows promise, particularly in enhancing privacy and reducing bandwidth usage, its current form requires significant advancements to effectively manage data heterogeneity and scalability challenges in the medical imaging domain.

#### 5.4.3 Comparative analysis

It is crucial to emphasize that the nature and scope of our research significantly differ from traditional studies that propose novel solutions. Our work is fundamentally an experimental survey focused on elucidating and quantifying the performance degradation in federated learning due to data heterogeneity among clients. This aspect has been theoretically discussed in numerous studies, but we have taken a step further by practically implementing and conducting a detailed experimental survey.

- Nature of the Study: Our research does not introduce a new model or solution but provides a comprehensive experimental investigation into an existing problem. This approach is distinct from most studies that propose new models or algorithms for comparison.
- Focus on Data Heterogeneity: The primary aim is to highlight the challenges and performance issues in federated learning due to data heterogeneity across clients. This has been a theoretical discussion in many studies, but our work brings practical insights and empirical data to the forefront.
- Unique Dataset Utilization: Unlike previous works that predominantly used predefined, built-in datasets like CIFAR-10 or MNIST, our study employs a dataset that has not been traditionally used in federated learning research focused on data heterogeneity. This choice of dataset adds novelty to our approach and provides fresh perspectives on the challenges in federated learning.
- Thorough Data Preparation for Federated Learning: We have undertaken extensive data preparation specific to the needs of federated learning experiments. This level of detailed preparation sets our work apart from others who might not have delved as deeply into the nuances of data readiness for federated learning scenarios.

Considering these distinctions, a direct comparison with previous models or solutions, as suggested by the reviewer, might not accurately reflect the contribution and uniqueness of our work. Our experimental survey serves as a critical bridge between theory and practice, offering new insights into a known theoretical problem by employing a dataset not previously used in this context. This approach enriches the federated learning research landscape by providing empirical evidence and detailed analysis, thereby contributing to a more nuanced understanding of the challenges in this domain.

We have provided the comparative analysis in Table 9. In comparing the proposed study with the works of Zhao et al. and Zhu et al., several vital aspects highlight the distinctiveness and advantages of each approach. The proposed study employs the Federated Averaging (FedAvg) algorithm, enhanced by the integration of advanced deep learning models such as Convolutional Neural Networks (CNN) and Multi-Layer Perceptrons (MLP). This combination is particularly adept at handling complex datasets, such as medical images, enabling the capture of intricate patterns in the data. In contrast, Zhao et al.’s study utilizes the FedAvg algorithm but does not specify the incorporation of advanced neural networks, which might limit the depth of their data analysis. Zhu et al.’s approach diverges significantly as it focuses on survey analysis, thus lacking in the practical application of federated learning techniques. The proposed study introduces dataset partitioning to simulate real-world data heterogeneity, enhancing the relevance and applicability of its findings to practical scenarios. Zhao et al. adopt specific sampling methods that, while effective, may not comprehensively represent the challenges of data diversity encountered in real-world applications. Zhu et al., focusing on conceptual review, do not delve into the intricacies of data preprocessing, a crucial step for the practical implementation of federated learning models.

**Table 9:** Comparative analysis.

Aspect	Proposed Study	[11]	[47]
Techniques Used	FedAvg with CNN, MLP	FedAvg	Survey Analysis
Preprocessing Techniques	Dataset partitioning	Specific sampling	Conceptual Review
Type of Dataset Used	COVIDx CXR-3	Predefined dataset	General IoT Data
Evaluation Measures	Global Accuracy, Model Convergence	Model Accuracy	No
Benefits	Emphasizes data heterogeneity challenges	Addresses fairness	Comprehensive survey
Multiple Datasets	Utilized more datasets	Limited to one dataset	Lacks practical implementation
Scalability	Yes	No	No

A significant strength of the proposed study lies in its use of the COVIDx CXR-3 dataset, a current and highly relevant dataset for COVID-19 medical imaging, reflecting the urgency and applicability of the study to contemporary healthcare challenges. Zhao et al.’s reliance on predefined datasets might not capture the complexity and variability inherent in newer, real-world datasets like COVIDx CXR-3. Zhu et al. employ general IoT data, which lacks the specificity required for a focused study in medical imaging. The proposed study adopts a comprehensive approach by evaluating global accuracy and model convergence. This dual focus provides a complete picture of the model’s performance, particularly in its application to real-world data scenarios. Zhao et al.’s study centers on model accuracy, which, while important, may not fully encapsulate the dynamics of performance in federated learning, especially in heterogeneous data environments. Zhu et al.’s study does not incorporate practical evaluation measures, limiting its utility in assessing the effectiveness of federated learning models.

The proposed study is particularly notable for its emphasis on data heterogeneity in federated learning, a crucial issue in practical applications, especially in healthcare settings. While addressing fairness in federated learning, Zhao et al.’s work does not directly confront the technical challenges posed by data heterogeneity. Zhu et al. provide a comprehensive survey, but it lacks the practical insights that empirical studies offer. The proposed study utilizes multiple datasets, showcasing its adaptability and robustness across various data types and distributions. This contrasts Zhao et al.’s study, which is limited to one dataset and does not demonstrate the model’s effectiveness across different scenarios. Zhu et al.’s work, lacking in practical implementation, does not provide evidence of effectiveness across multiple datasets. Furthermore, the proposed study demonstrates scalability, an essential feature for applications in dynamic scenarios like a pandemic, a feature not explicitly focused on in the other two studies. The proposed study stands out for its practical application, use of advanced techniques, focus on real-world challenges like data heterogeneity, and comprehensive evaluation measures. It addresses theoretical concepts and validates them through empirical data, making it a significant and relevant contribution to federated learning in medical imaging.

Our work stands out from existing efforts in several distinct ways.

1. **Experimental Focus**: Unlike the general survey approach taken by [47], our study delves into an experimental exploration, providing concrete empirical evidence on how data heterogeneity affects FL performance in medical imaging. This experimental approach allows us to support the theoretical discussions in [47] and extend them with practical findings and insights specific to the medical domain.
2. **Unique Dataset Application**: no prior FL research has utilized the COVIDx CXR-3 dataset to evaluate non-IID data setups. This gap in the literature signifies the unique contribution of our work, as we are the first to explore the implications of data heterogeneity on FL algorithms using this specific, highly relevant dataset. The COVIDx CXR-3 dataset, with its rich diversity and real-world applicability to current health challenges, offers a novel avenue for understanding and improving FL in the fight against COVID-19.
3. **Contribution to COVID-19 Research**: Our work is particularly timely and relevant, given the ongoing global health crisis. By focusing on COVID-19 medical images, we contribute to the body of knowledge in a way that directly impacts public health responses and strategies, offering insights into leveraging FL for pandemic-related medical imaging analysis.

## 6 Potential solutions to mitigate data heterogeneity

In the context of FL, data heterogeneity poses challenges due to the variance in data distribution across different clients. Below are some methods to mitigate these issues:

- Advanced Aggregation Methods
  In Federated Learning (FL), different clients may have data with different characteristics. A naive approach like simple averaging of model parameters might not work well in such scenarios. Advanced aggregation techniques like robust aggregation focus on minimizing the impact of outlier clients whose data significantly differ. For example, algorithms like FedMed or Byzantine-robust aggregation methods can give different weights to different clients based on the quality or representativeness of their updates. The goal is to derive a global model that generalizes well while reducing the adverse effects caused by the heterogeneity among clients.
- Transfer Learning
  Transfer learning leverages a model pre-trained on a related but potentially different task to accelerate and improve the model training process in FL. This is especially useful when some clients have small or less representative datasets. Imagine a federated learning system for medical diagnostics involving various hospitals. A model pre-trained on a large, centralized dataset of X-ray images can be fine-tuned locally at each hospital. This allows each local model to adapt more quickly and accurately to the specific types of data seen at each hospital
- Personalized FL
  Traditional FL aims to create a single, global model that works well across all clients. However, sometimes the need for personalization outweighs the desire for a global solution. Personalized FL targets training local models that are tailored to each client’s unique data distribution. For example, consider a federated learning system for recommendation algorithms on smartphones. Each smartphone could have a model that is personalized to its user’s behavior, thus improving the quality of recommendations for each individual user.
- Ensemble Techniques
  Ensemble methods involve combining multiple models to improve performance and robustness. In the context of FL, models trained on different clients can be aggregated into an ensemble model, where each local model ‘votes’ to make a final decision. This approach captures the diversity in data patterns across clients, potentially offering more accurate and robust predictions than any single model.
- Client Selection Mechanisms
  Not all clients contribute equally valuable updates in federated learning. Client selection mechanisms aim to pick the most informative or reliable clients for training in each round. For instance, some methods might select clients based on the diversity of their data, their past contributions to model improvement, or even their computational capabilities. This selection ensures that the global model’s training is both efficient and effective.
- Knowledge Graph for Federated Learning
  A knowledge graph can be used to map out the relationships and attributes of different clients and their data in FL. This meta-information can assist in the aggregation process, client selection, or even in the training of more specialized local models. For example, in a federated learning setup across multiple hospitals, a knowledge graph might contain information about each hospital’s specializations, the demographics they serve, etc. This knowledge can be used to inform a more intelligent aggregation of models.
- Straggler Mitigation Techniques
  Stragglers in FL are clients that are slow to train models or send updates, delaying the entire process. Techniques such as gradient sparsification can reduce the amount of data that needs to be transmitted, thereby speeding up the slowest participants. Asynchronous updates allow faster clients to continue without waiting for the slower ones.
- Learning Rate Schedules
  Different clients in an FL setting may require different learning rates due to the heterogeneity in their data. Adaptive learning rates, like those offered by algorithms like Adam, can automatically adjust the learning rate for each client or even each feature, optimizing the speed of model convergence across diverse data distributions.
- Context Awareness
  This involves taking into account additional information about each client’s current situation, like location, time, or device status. Context awareness can guide the training process, client selection, or model deployment in a way that is more adaptive to real-world conditions. For example, a weather prediction model in an FL system could give more weight to clients from regions currently experiencing severe weather events, making the model more responsive to immediate needs.
- Participant Selection
  In some FL applications, it might be necessary to select participants based on attributes other than their data. These could include their available computational resources, network latency, or even the trustworthiness of the client. For example, in a sensitive application like healthcare, you might only want to include clients (e.g., hospitals or health centers) that meet specific compliance standards.
- Selective Updating
  Instead of updating the local models on all clients after each round of federated learning, selective updating chooses only a subset of clients for updating. This method can focus on clients where the model performs poorly or where the data has substantially changed. For instance, in a text prediction application, clients that start using a new slang or terminology may be selectively updated to adapt to these changes.
- Contextual Client Selection
  In this strategy, the context in which a client operates (e.g., geographical location, time of activity, etc.) influences their inclusion in training rounds. For example, an FL model for e-commerce might update more frequently for clients (or stores) that experience high-traffic during holiday seasons, thereby capturing fast-changing trends. Contextual client selection ensures that the federated model remains adaptive to shifts in the environment or user behavior.

## 7 Conclusions

This research represents a pivotal advancement in comprehending the influence of data heterogeneity on federated learning (FL) algorithms, with a particular focus on medical imaging data. We meticulously developed a thorough data preparation and preprocessing method explicitly tailored for FL. This approach was adaptable, accommodating various numbers of clients, communication rounds, and dataset sizes, which established a robust groundwork for our experimental evaluations. Our methodology enabled us to create homogeneous (IID) and heterogeneous (non-IID) environments, mirroring real-world scenarios. This setup facilitated an in-depth Federated Averaging (FedAvg) algorithm assessment. One of the key findings of our study is the observable reduction in both the performance and accuracy of FL models as data heterogeneity increases. This outcome underscores the significant challenges encountered in FL deployments, mainly when data distribution is uneven across different devices. Such insights highlight the pressing need for targeted research efforts to develop strategies to enhance FL algorithm convergence in the face of data heterogeneity. Our study lays a solid foundation for future investigations in this domain, setting the stage for developing more sophisticated and resilient FL algorithms. These advanced algorithms are envisioned to be better equipped to handle the diversity and imbalances inherent in real-world data. The methodology we employed and the insights and results gleaned from our study make substantial contributions to the ongoing discussions and explorations in FL. They underscore the importance and urgency of further research and innovation in this rapidly evolving field, encouraging the scientific community to delve deeper and develop solutions that address these critical challenges.
